# Endocrine disruptors alter social behaviors and indirectly influence social hierarchies *via* changes in body weight

**DOI:** 10.1186/s12940-015-0051-6

**Published:** 2015-08-05

**Authors:** Benjamin Kim, Eliezer Colon, Shivansh Chawla, Laura N. Vandenberg, Alexander Suvorov

**Affiliations:** Department of Environmental Health Sciences, School of Public Health & Health Sciences (SPHHS), University of Massachusetts, 686 N. Pleasant Street, Goessmann 149A, Amherst, MA 01003 USA

**Keywords:** Body weight, Social status, Social dominance, Sociability, Endocrine disruption, Tetrabromobisphenol-A, TBBPA, 2,2,4,4-tetrabromodiphenyl ether, BDE-47, Bisphenol S, BPS

## Abstract

**Background:**

In humans, the causal link between socioeconomic status (SES) and body weight (BW) is bidirectional, as chronic stress associated with low SES may increase risk of obesity and excess weight may worsen career opportunities resulting in lower SES. We hypothesize that environmental factors affecting BW and/or social stress might reprogram physiological and social trajectories of individuals.

**Objectives:**

To analyze interactions between BW and social behaviors in mice perinatally exposed to one of several environmental endocrine disruptors.

**Methods:**

CD-1 mice were fed 0.2 mg/kg BW/day tetrabromobisphenol-A (TBBPA), 2,2,4,4-tetrabromodiphenyl ether (BDE-47), bisphenol S (BPS), or oil (vehicle) from pregnancy day 8 through postpartum day 21. Three male offspring (triad) from each litter were housed together until week 15 and subjected to a Sociability Test and Tube Tests. Cages were then rearranged so that animals of the same social rank from the four exposure groups were housed together in tetrads. Social hierarchy in tetrads was again analyzed by Tube Tests.

**Results:**

In Sociability Tests, the mean velocity of all exposed animals increased when they encountered a stranger mouse and less time was spent with conspecifics. BW and social dominance of animals in triads and tetrads were inversely associated. BDE-47 and BPS caused transient decreases in BW.

**Conclusions:**

Developmental exposure to environmental xenobiotics shifted behavior towards increased anxiety and decreased interest in social interactions. Our mouse model reproduces negative associations between social hierarchy status and BW. These results suggest that manipulation of BW by endocrine disruptors may affect social ranking.

**Electronic supplementary material:**

The online version of this article (doi:10.1186/s12940-015-0051-6) contains supplementary material, which is available to authorized users.

## Background

The worldwide burden of obesity has doubled since the 1980s and obesity has been described as a global epidemic with 36.9 % of males and 38.0 % of females worldwide classified as overweight or obese [1]. Obesity increases the risk of several debilitating comorbidities including diabetes, cardiovascular disease, and several forms of cancer [2]. It is the second leading cause of preventable death in the United States [3]. It is also associated with weight related social stigma [4].

The chronic stress of adverse social interactions may increase food consumption [5, 6] and result in increased risk of abdominal obesity [7]. For example, in mice, mild social stress accelerates food intake and body weight gain [5]. Further, social stress of subordination induces hyperphagia and exacerbates diet-induced insulin resistance and metabolic syndrome in adult mice [8]. These results reproduce the effects of low socioeconomic status on human health. In fact, stress favors emotional eating of high fat/high sugar foods, leading to increased risk of obesity in humans [9]. A cyclic model has been recently proposed to characterize a positive feedback loop wherein weight stigma begets weight gain through increased eating behavior [10]. This model may potentially explain the very modest progress achieved by numerous trials targeted at body weight management by pharmacotherapy interventions and lifestyle modifications [11], because positive feedbacks between energy imbalance, disruption of the neuroendocrine axes, and adverse psychological factors may result in a formation of a long term self-sustaining maladaptive trajectory. Experimental testing of the hypothesis linking body weight and social stress by positive feedbacks is therefore needed to advance our knowledge about the etiology of obesity and open the window for development of new and efficient therapeutic interventions. Laboratory animal based research may be especially useful for the study of the role of environmental factors in the epidemic of obesity. However, it is not clear if causal links between different elements of the “vicious cycle” can be modeled in laboratory rodents.

We hypothesize that environmental factors, positively affecting BW and/or negatively affecting social status, might cause reprogramming of physiological and social trajectories of affected individuals. Endocrine disrupting chemicals (EDCs) are one plausible environmental trigger. Evidence from both epidemiological and animal studies (reviewed by [12, 13]) suggest that many EDCs disrupt energy metabolism and BW in exposed subjects [14]. Some environmental chemicals are also known as potent modifiers of social behavior. For example, increased lead exposures have been associated with a number of well-documented social consequences including violence and teenage pregnancy rates [15]. Different aspects of social behavior are impaired in laboratory rodents exposed to diverse EDCs including PCBs [16], BPA [17, 18], mercury, cadmium [19], and vinclozolin [20], among others [21].

In this study, we analyzed the effects of perinatal exposure to EDCs on social behavior and on the interaction between BW and social stress in mice. We selected for our study three compounds: 2,2,4,4-tetrabromodiphenyl ether (BDE-47), one of the most prevalent congeners of polybromodiphenyl ethers (PBDEs) found in maternal milk and fetal samples [22–27]; tetrabromobisphenol-A (TBBPA), a chemical substitute of PBDE; and bisphenol-S (BPS), a substitute of bisphenol-A (BPA). We explored the links between developmental EDC exposures, social behavior, weight and social stress in CD-1 mice. As a measure of social stress, we used social rank within groups of cohabited mice as subordination is a relevant model for investigating the behavioral, neural and endocrine correlates of chronic stress [28, 29]. In identification of social ranks, we capitalized on the validated Tube Test, a reliable measure of dominance ranking [30]. We report effects of these EDCs on body weight, sociability, social status, and interactions between these factors.

## Material and methods

### Animals and treatment

8-week old male (30–35 g) and female (27–30 g) CD-1 mice were obtained from Charles River Laboratories (Kingston, NY, USA) and housed in a temperature (23 ± 2 °C), and humidity (40 ± 10 %) controlled environment, with a 12-h light/dark cycle, and food and water available *ad libitum*. After 3 days of acclimation, animals were bred and the day of vaginal plug detection was considered pregnancy day 1. Dams were assigned to one of four treatment groups (*n* = 5 per group) based on weight match and exposed to tocopherol stripped corn oil (MP Biomedicals, Solon, OH) or 0.2 mg/ml solutions of TBBPA (Sigma-Aldrich, 97 % purity), BDE-47 (AccuStandard, Inc., New Haven, CT; 100 % purity) or BPS (Santa Cruz Biotechnology, Dallas, TX, 99 % purity) in tocopherol stripped corn oil daily from pregnancy day 8 through postpartum day 21; females were fed 1 μl/gram BW from the tip of a pipette, resulting in exposure of 0.2 mg/kg BW/day. Dams were allowed to deliver naturally, and the litters were not culled to maintain consistency of nutrient distribution among the same number of fetuses/pups at pre- and postnatal periods and avoid catch-up growth. Dams and pups were kept together until weaning on PND 21, when male and female pups were separated. All procedures met the guidelines of the National Institutes of Health Guide for the Care and Use of Laboratory Animals and this study was approved by the Institutional Animal Care and Use Committee at University of Massachusetts, Amherst.

### General outline of behavior testing

**Fig. 1:**
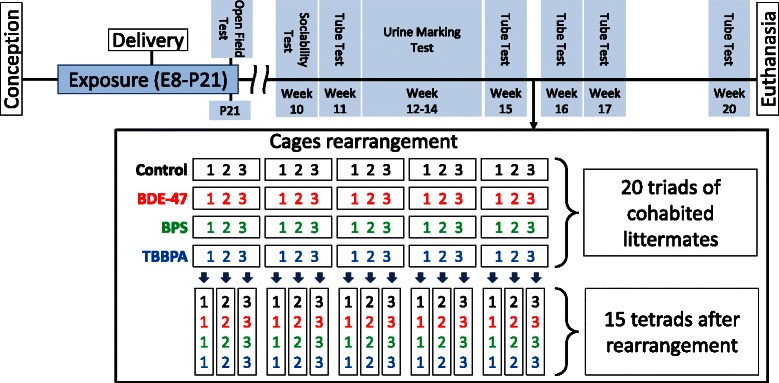
Scheme of experimental design including sequence of exposure and behavior testing. The scheme in the box illustrates cages rearrangement at the end of postnatal week 15. Numbers in blocks indicate social ranks of animals determined by Tube Test in triads on week 15

One male pup per litter was randomly selected and tested for spontaneous motor activity in the Open Field test on PND 21. After weaning, three male pups (triad) from each litter were randomly selected and housed together until postnatal week 15. The behavior test sequence for these males is shown in Fig. 1. Briefly, triads from the same litter were housed together until the end of postnatal week 15. On week 10, males were tested on the sociability apparatus, and on week 11 they were subjected to the Tube Test to measure social hierarchy within the triad. On weeks 12 through 14, a Urine Marking Test was performed to further reveal social hierarchy inside of each triad. To check for the stability of social structure we repeated the Tube Test on week 15. Immediately after this, cages were rearranged. Animals of the same social rank (i.e., dominant, middle, subordinate) from the four different exposure groups (control, TBBPA, BDE-47, BPS) were housed together in tetrads. Thus, 20 cages containing 3 littermate mice each were rearranged into 15 cages with 4 mice in each. Social hierarchy in tetrads was analyzed by Tube Test on postnatal weeks 16, 17, and 20. All behavioral tests were done between 9 a.m. and 12 p.m. by personnel blinded to exposure groups.

### Open field test

One male pup per litter was randomly selected on PND21 and assigned for evaluation of spontaneous motor activity. The testing procedure was conducted as described in our previous paper [31]. In short, pups were placed in the corner of an open field apparatus. The apparatus consisted of a 45 cm × 45 cm base and a 40-cm high enclosure. Movements were filmed for 5 min beginning as soon as the animal was placed in the apparatus. The camera, mounted directly above the apparatus, was attached to a computer running the EthoVision XT10 software (Noldus, Wageningen, Netherlands) which tracked the center of the animal and recorded its displacements. The following parameters were analyzed: total distance travelled, mean speed, and cumulative duration of stay in the center of the apparatus (20 × 20 cm).

### Sociability analysis

To assess sociability and the preference for social novelty in male mice, we used the Three-Chamber Social Approach Test described elsewhere [32, 33]. Briefly, the apparatus consists of a 25x40x60 cm (HxWxL) box with partitions separating the box into three equal-sized chambers. The partitions have openings that allow the animal to move freely from one chamber to another. The openings have sliding doors. The test was performed in 3 consecutive steps: 1) the animal was placed in the middle chamber with the sliding doors closed to allow it to explore the chamber for five minutes; 2) an unfamiliar male mouse of the same age and strain (stranger 1) was placed inside a small wire cage in one of the side chambers, an identical empty wire cage was placed in the opposite chamber, the sliding doors were then raised, allowing the test subject to move freely throughout all three chambers over a 10-min test session; 3) stranger 1 (now familiar mouse) remained in his wire cage on one side of the apparatus and a new unfamiliar male mouse (stranger 2/novel mouse) was placed in the wire cage on the opposite side, and the test subject was allowed to move freely over another 10-min test session. Movements of the test subject were recorded throughout the duration of each step by the camera attached to a computer running the EthoVision XT10 software. Location of the stranger mouse and the empty wire cage was alternated between left and right chambers on consecutive sessions. Measures calculated for each chamber and for the 5 cm zone surrounding each wire cage included: total distance moved, mean velocity, mean angular velocity, and total time spent in the chamber/zone. Further a Sociability Index (SI) and Social Novelty Preference Index (SNI) were calculated using equations described elsewhere [34]. SI for the second stage of the test = (time spent in side chamber with stranger - time spent in empty side chamber)/(time spent in side chamber with stranger + time spent in empty side chamber); SNI for the third stage of the test = (time spent in side chamber with novel mouse - time spent in side chamber with familiar mouse)/(time spent in side chamber with novel mouse + time spent in side chamber with familiar mouse).

### Analysis of social hierarchy

To analyze relations of social dominance/subordination between cohabited male mice, we used the Tube Test as described elsewhere [30]. Briefly, the apparatus consists of a 30 cm long transparent plastic tube with an inner diameter of 32 mm, mounted horizontally on a stand so that both ends of the tube freely hang in the air 20 cm above the bench surface. Two mice from the same cage were simultaneously loaded into opposite ends of the tube. The trial continues until one of the mice is pushed out of the tube. The duration of the test was recorded. The winning mouse is considered the dominant animal. Within each cage, paired encounters were staged such that each mouse encountered every other mouse of the group only once; in total, three pairs were tested per triad and six pairs were tested per tetrad. A Urine Marking Test was also performed as a measure of social hierarchy [30]. In all cases of clear subordinate/dominant patterns of urine marking, the results matched tube test conducted the week prior. Therefore, we used the Tube Test to measure social dominance.

### Statistical analysis

All statistical analyses were performed using SPSS Statistics 22 software. Equality of variances in exposure groups was analyzed using Levene’s test. To analyze the behavior of mice in the side chambers of the sociability apparatus during the second and third steps of the test, a mixed ANOVA analysis was conducted using the behavior readings from two chambers as the within-subject factors and group of exposure as the between-subject factors. A *T*-test was further used to identify if behavior parameters and BW were affected by exposure. *P*-values for behavior parameters were adjusted using the FDR method [35] to correct for multiple comparisons. General linear model (GLM) was used to analyze changes in BW in relation to xenobiotic exposure with litter size and exposure group as covariates, to study the interaction of litter size with performance in behavior tests, and to analyze effect of exposure, litter size and BW/weight rank on social rank in tetrads. Litter ID was also included in GLM to evaluate effect of litter other than litter size on social rank in tetrads. Spearman correlation (*r*_*s*_) was used to analyze the association between social ranks, test duration, weight ranks of animals in their triads or tetrads and weight gain between consecutive Tube Tests. To evaluate stability of social hierarchy in these groups of mice, we analyzed agreement of ratings at different time points using Kappa statistics.

## Results

### Litter size and BW

There was no significant difference in mean litter sizes between exposure groups (data not shown). However, variation of litter sizes was significantly higher in TBBPA group in comparison with every other groups (Levene’s test for Equality of variances, *p* <0.05). While litter sizes had ranges of 11–15 pups in Control, BDE-47 and BPS groups, in TBBPA group five litters had the following numbers of pups: 5, 7, 11, 14, 15. No weight differences were observed for control and exposed dams throughout pregnancy and lactation. There were also no significant changes in average pup weight adjusted for litter size at birth.

**Fig. 2:**
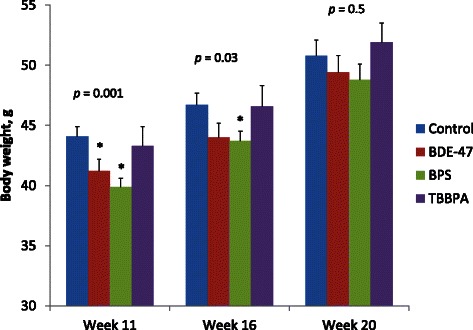
Body weight of male pups in relation to exposure group (Mean ± SE). All *p*-values are for general linear model with litter size as covariate. Asterisks indicate significant difference (*T*-test *p* < 0.05) with control

On the day of the first Tube Test, the weight of the male mice was significantly lower in groups exposed to BDE-47 and BPS compared to controls (Fig. 2). Both exposure group and litter size were significant covariates for BW in general linear model (GLM, *p* = 0.001 and *p* < 0.001 respectively). Effect of exposure group on BW remained significant on week 16 (GLM, *p* = 0.03) but disappeared by week 20 (GLM, *p* = 0.5) while litter size effect remained significant throughout the experiment (GLM, *p* < 0.001 and *p* = 0.001 for week 16 and 20, respectively).

### Spontaneous motor activity

There were no significant differences between control and exposed pups in any of the parameters measured in the Open Field Test (data not shown). Also, no interaction was found between any of the motor activity parameters and litter size.

### Sociability

At stage I of the sociability test, BPS exposed mice had significantly higher mean velocity of movement compared to controls (Additional file 1); no differences were observed for TBBPA or BDE-47 treated males at this stage.

**Fig. 3:**
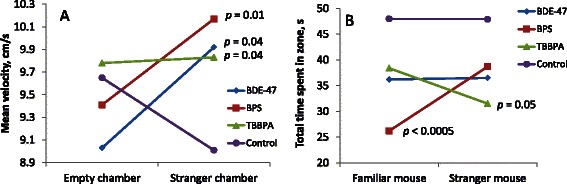
Behavior performance of control and exposed animals in Sociability Test: **a** – velocity of test subjects in the empty chamber and chamber with stranger mouse (mixed ANOVA *p* = 0.025); **b** – time spent by test subject in the old stranger and new stranger wire cage zones (mixed ANOVA *p* = 0.33). All *p*-values shown on the plot are for FDR adjusted *T*-test

At stage II, there was a significant interaction between within-subject repeated measures of velocity in the side chambers and exposure group (*p* = 0.025). Mean velocity of control animals decreased as they approached the stranger mouse, whereas mean velocity increased in all exposed animals, although to different degrees depending on treatment - see Fig. 3a. The mean velocity of all exposed animals in the chamber with the stranger mouse was significantly higher than the mean velocity of controls (Fig. 3a): mean velocity of test subjects exposed to BDE-47, BPS, and TBBPA was 9.7 %, 12.5 % and 8.6 % higher than controls, respectively. Velocity of these animals was also higher in the middle chamber, but this difference was only significant in the TBBPA group (FDR adjusted *p* < 0.05). SI was not significantly different between exposure groups. SI for Control, BDE-47, BPS, and TBBPA groups were 0.42 ± 0.08, 0.47 ± 0.09, 0.42 ± 0.09 and 0.44 ± 0.06, respectively (Mean ± SE).

At the stage of social novelty testing (stage III), no significant interaction was found between within-subject repeated measures in chambers with familiar and stranger mice and exposure group. However, all exposed animals spent less time with both the familiar and novel mice (Fig. 3b), although the BDE-47 group was not significantly different from control. In this stage of the test, animals exposed to BPS spent less time with the familiar mouse as indicated by significant differences in the total distance traveled, time spent in the chamber, and time spent in the 5 cm zone surrounding the wire cage with the familiar mouse. TBBPA exposed animals displayed reduced interest in social novelty as they moved significantly shorter distances and spent less time in the 5 cm zone of wire cage with the novel mouse. In total, the time spent by test subjects exposed to BDE-47, BPS and TBBPA in the 5 cm zone with either the familiar or novel mouse decreased 22 %, 35 % and 27 %, respectively. SNI was not significantly different between exposure groups. SNI for Control, BDE-47, BPS, and TBBPA groups were −0.06 ± 0.05, 0.06 ± 0.07, 0.06 ± 0.06 and −0.08 ± 0.03 respectively (Mean ± SE). No interaction was found between behavior measures in the Sociability Test and litter size.

### Relation of social rank and BW in triads of littermates

Within each triad, three pairwise tube tests were performed, allowing for quantitative measurements of interactions between each pair. At week 11, no significant differences were observed in the duration of the test based on exposure groups (data not shown). Each animal was assigned a social rank from 1 to 3, where 1 is the most dominant animal that won all pairwise social encounters and 3 is the most subordinate mouse that lost all encounters. Surprisingly, social dominance was inversely related to BW [rank 1 weight: 40.6 ± 0.8 g; rank 2: 42.3 ± 0.9 g; rank 3: 42.8 ± 0.8 g; (Mean ± SE)], although this relationship was not statistically significant.

**Fig. 4:**
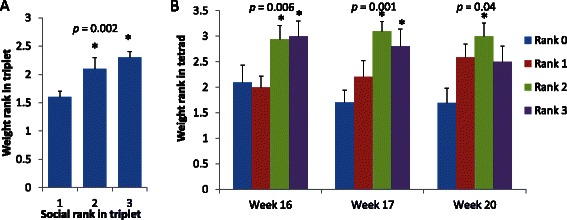
Weight ranks of mice with different social rank in triads of littermates on postnatal week 11 (**a**) and in tetrads composed of animals from different exposure groups on weeks 16, 17 and 20 (**b**). The smaller value of social rank indicate more dominant status, smaller value of weight rank indicate lighter animal. All *p*-values are for Spearman correlation, asterisks indicate significant difference (*T*-test *p* < 0.05) with dominant animals

We next hypothesized that relative weight of the subject animal in relation to other animals, rather than absolute weight, influences social hierarchy. We ranked animals by assigning weight rank 1 to lightest animals, weight rank 2 to animals having medium weight and weight rank of 3 to the heaviest animals in each triad. Weight ranks of subordinate animals and animals with intermediate social hierarchy were significantly higher than dominant animals (Fig. 4a) and association of weight rank with social rank was significant (*r*_*s*_ = 0.4, *p* = 0.002). There were no significant correlations between body weight gain between Tube Tests and social ranks. Stability of social ranking was assessed by comparing results of Tube Tests performed on weeks 11 and 15. Consistent social rankings were observed over this period of time (Kappa = 0.45, *p* < 0.001).

### Relation of social rank, BW and exposure group in tetrads

Social ranking in triads from week 15 was used to rearrange animals in tetrads with one animal from each treatment group. Animals were given three days to establish a new social hierarchy and then subjected to the Tube Test. The duration of the first Tube Test after rearrangement negatively correlated with the initial social rank of the animals, i.e., animals established as more dominant while in their triads completed the Tube Test slower in new tetrads compared to animals ranked as subordinate (*r*_*s*_ = −0.35, *p* = 0.001). Based on the results of Tube Tests, each animal was assigned a social rank ranging from 0 (most dominant) to 3 (most subordinate).

Throughout the experiment, association of BW (weight rank within the tetrad) with social rank persisted: *r*_*s*_ = 0.35, *p* = 0.006 for week 16; *r*_*s*_ = 0.42, *p* = 0.001 for week 17; and *r*_*s*_ = 0.27, *p* = 0.041 for week 20 (Fig. 4b). On weeks 16 and 17, BWs of the two most subordinate ranks were significantly heavier (*p* < 0.05) than weights of the most dominant animals. On week 20, only mice having a social rank of 2 were significantly different from the most dominant animals. Exposure group, litter, and litter size effects on social ranking in all Tube Tests performed in tetrads were not significant in GLM with BW or BW rank as covariates. There was no significant correlation between body weight gain between Tube Tests and social ranks. Agreement between tests conducted on weeks 16 and 17 and 17 and 20 was as follows: Kappa = 0.44, *p* < 0.001 and Kappa = 0.31, *p* < 0.001, respectively.

## Discussion

The four major findings of this study are as follows: 1) developmental exposures to three environmental chemicals (BDE-47, BPS and TBBPA) affect social behavior in mice; 2) two of these substances (BDE-47 and BPS) have lasting effects on BW; 3) BW is a predictor of social hierarchy rank, regardless of environmental chemical treatment; and 4) the methodology used here may be useful to analyze relationships between BW and social status and dissect the interplay between environmental chemical exposures, social behavior and metabolic endpoints.

### Relevance and applicability of exposure levels to humans

In our previous study [31], exposure of pregnant rats to 0.2 mg/kg BW of BDE-47 resulted in 234.3 ng BDE-47/g lipid in adipose tissue of dams; these concentrations are comparable with that of the North American human population (mean for adipose tissue = 399 ng/g lipids) [36]. Given that the half-life of BDE-47 is around 10 times shorter in mice [37] than in rats [38], BDE-47 doses used in this study should be considered low and environmentally relevant. Data about BPS and TBBPA exposures in the general population remain scarce but are likely somewhat lower than those used here. Based on urinary concentrations, the median daily intake of BPS was estimated as 1.67 μg/person in Japan and 0.339 μg/person in the US [39]. Studies estimate daily exposure of toddlers to 0.2 ng/kg BW/day TBBPA via dust [40]. In human adipose tissue, average concentrations of TBBPA (mean+/−SD) were 0.048 ± 0.102 ng/g lipid [41]. These low concentrations of TBBPA are a result of relatively little bioaccumulation and the relatively short half-life of this substance in mammals (<3 days in rats [42], 6.6 days in humans [43]).

### Exposure to EDCs and social behavior

Our results revealed effects of developmental exposures to three environmental chemicals on social behavior in male mice assessed seven weeks after exposure. When subjects encountered a stranger mouse, animals of all three exposed groups moved faster than control animals. This behavior is likely a result of increased anxiety. At the third step of the test, when a novel mouse was added to a side chamber, all exposed mice demonstrated decreased interest in social interactions compared to control animals, spending less time with both conspecifics. This behavior may also be explained by increased anxiety and resembles reduced interest in social contacts in patients with autism spectrum disorder (ASD) [44]. Though statistically significant, the differences we observed in the behavior of exposed and control animals were not drastic, although the shift in the behavior profile of exposed mice is concerning. The prevalence of neurodevelopmental disorders has risen dramatically in children in the United States in recent decades [45]. The role of environmental chemicals in this epidemic is poorly understood, however in a meta-analysis, positive associations were found for ASD and ADHD in relation to exposure to a broad range of xenobiotics including PBDE and BPA [46]. Human data linking brominated flame retardant exposures with altered behavior and neurodevelopmental disorders has also recently begun to emerge [47–50]. A recent study in rodents revealed that both TBBPA and BPS altered maternal behaviors in mice exposed during pregnancy and lactation; specifically, dams exposed to TBBPA had decreased latency to retrieve pups [51], consistent with TBBPA behavioral effects observed in the developmentally exposed males in the current study.

It was recently shown that both ASD and ADHD exist as the extreme of a behavioral continuum [52, 53]. Shifts in the distribution of these quantitative behavior traits due to the developmental toxicity of environmental EDCs may result in a substantial increase of marginal phenotypes, recognized by health professionals as an epidemic of neurodevelopmental disorders. Our data, combined with data from other studies, support a link between environmental chemical exposures and shifts in the distribution of behavioral phenotypes.

### Exposure to EDCs, BW and social rank

Exposure group was a significant covariate for BW (after adjustment for litter size) on week 16 and 17 (but not 20), with decreased BWs observed in males exposed perinatally to BDE-47 and BPS. Previous literature indicates that these substances are potent modifiers of BW and growth, although the direction of effect varies due to the developmental windows, dosing protocols, and models. Developmental PBDE exposures resulted in increased BW in rodents [31, 54] and growth in birds [55]. However, other studies have shown growth suppression by PBDEs in rodents, amphibians, and fish [56–58]. Several studies report positive associations between PBDE concentrations and BMI in nursing women [59–61] whereas others report inverse associations between prenatal PBDEs and birth weight [62–64]. We are aware of only a single study that has examined the effects of BPS on BW; male zebrafish developmentally exposed to 100 μg/l of BPS had significantly decreased body lengths and weights compared to controls [65].

In our experiments, we found a somewhat counterintuitive negative association between social dominance and BW in triads of cohabited littermates and tetrads comprised of animals from different exposure groups; mice with higher BW had lower social ranks. These relations resemble the association between low socioeconomic status (SES) and higher risk of obesity in human populations [66, 67]. SES is an important determinant of BW as people with low incomes generally have limited access to healthy food, healthy lifestyles, and health related knowledge/skills. Furthermore, the chronic stress associated with low SES may trigger emotional eating of high calorie foods leading to increased risk of obesity [9]. Excess weight may have detrimental effects on career opportunities [68, 69] *via* decreases in health quality or weight stigmatization [70]. Negative effect of increased weight on “social success” was reproduced in our experiment with mice: four animals of the same social rank were placed together in tetrads and allowed to rebuild social hierarchy *de-novo*. This design allowed us to evaluate if BW is a predictor of social rank. Given that the BW/social rank association was significant in as little as three days after arrangement of tetrads, we conclude that BW may be a causative factor for social ranking.

**Fig. 5:**
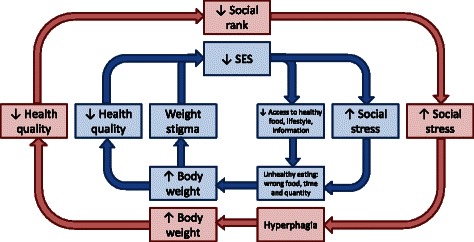
Hypothetical scheme of positive feedback loop linking social status of an organism with its body weight. The inner blue cycle depicts possible elements and causative links in human populations. The outer red cycle illustrates our animal model

It is likely that a cyclic model emphasizing a positive feedback loop between weight stigma, increased eating behavior, and weight gain [10] depicts only part of more complex relations, shown in Fig. 5. Of all these causative links potentially important for humans, likely only a few apply to our mouse model, i.e., the animals in our experiments all have equal access to food and an identical lifestyle (with the exception of chemical treatment). It is also unlikely that mice apply a stigma to overweight cagemates. However, it was shown previously that stress of subordination induces hyperphagia [8, 71], which may result in heavier weights of low-ranking animals. Additional studies are needed to test this hypothesis further. It is also possible that excess weight results in a decrease in health quality, which in turn determines social hierarchy rank. Finally, the possibility that coordination of BW and social rank may be determined by some unknown factor cannot be dismissed.

Negative associations between BW and social dominance that were observed in this study may seem counterintuitive, as it is much easier to comprehend that heavier, bigger animals would have higher odds of winning in social encounters. Positive associations between BW and social dominance have been observed in a wide range of wildlife species [72–75], although it is not a rule [76]. Wild populations are usually genetically heterogenic and have limited resources. The mouse strain used in our experiment, although outbred, has much lower genetic and morphological variability than wild animals [77]. Housing in standard conditions with *ad libitum* food does not constrain physiological development. Thus, we assume that major differences in weight observed in our experiments are due to adipose tissue, accumulation of which probably does not improve chances of winning in social encounters. Additional studies are needed to collect further information on the relationship between body composition (% body fat) and social structure in our mice.

After mice of the same social rank from different litters and exposure groups were placed together in tetrads, analysis of new social ranking revealed the same negative association between social rank and body weight; this association was significant throughout the experiment. Because all 4 animals that were placed together in these tetrads had the same social rank when they were placed together, the new social hierarchy was rebuilt *de-novo*. This design allowed us to evaluate if body weight is a predictor of social rank (rather than the opposite). Given that body weight/social rank association was significant in as little as three days after arrangement of tetrads, we conclude that body weight may be considered a causative factor for social ranking.

Our experiments revealed no significant relationships between perinatal exposure group and social rank. Thus, although xenobiotics used in this study altered the social behavior of mice in the sociability apparatus, exposures did not have any direct effect on social dominance. However, exposures may influence social ranks via indirect effects on BW. In the current study, developmental exposure to BDE-47 and BPS resulted in transitional decreases in BWs, thus increasing their odds of gaining dominant social status. The importance of these results lies in the principal ability to manipulate the social rank of mice by developmental exposures to substances that modulate BW. The possibility that environmental xenobiotics could affect social success of an individual is concerning and calls for an introduction of a social dimension in experimental toxicological research.

## Conclusions

We have shown that developmental exposure to three ubiquitous environmental xenobiotics can shift quantitative behavior traits in mice towards increased anxiety and decreased interest in social interactions. Second, we have developed a mouse model to simulate the positive feedback loop between BW and social status - a putative mechanism influencing maladaptive physiological and social trajectories in human populations. We have shown that manipulation of BW by EDCs may affect social rank of animals. We believe that this model may become an important tool for the understanding of the complex interplay of behavioral, social, environmental, and metabolic factors in the development of the obesity epidemic.

## Additional file

Additional file 1: **Performance in sociability test of male mouse offspring exposed perinatally to different plastic additives.** Test was done on postnatal week 10. Excel spreadsheet with data (mean, SE, FDR corrected *p*-value) on behavior parameters, readouts of sociability test, performed on postnatal week 10 with male mice offspring exposed perinatally to one of 4 testing conditions (vehicle only, BDE-47, TBBPA, BPS). (XLSX 17 kb)

## Abbreviations

ADHD: Attention deficit hyperactivity disorder
ASD: Autism spectrum disorder
BDE-47: 2,2,4,4-tetrabromodiphenyl ether
BMI: Body mass index
BPS: Bisphenol S
BW: Body weight
EDC: Endocrine disrupting chemical
FDR: False discovery rate
GLM: General linear model
PCB: Polychlorinated biphenyl
PND: Postnatal day
SES: Socioeconomic status
TBBPA: Tetrabromobisphenol-A
